# PmtA Regulates Pyocyanin Expression and Biofilm Formation in *Pseudomonas aeruginosa*

**DOI:** 10.3389/fmicb.2021.789765

**Published:** 2021-11-15

**Authors:** Amy V. Thees, Kathryn M. Pietrosimone, Clare K. Melchiorre, Jeremiah N. Marden, Joerg Graf, Michael A. Lynes, Michele Maltz-Matyschsyk

**Affiliations:** Department of Molecular and Cell Biology, University of Connecticut, Mansfield, CT, United States

**Keywords:** metallothionein, pyocyanin, biofilm, antibiotic resistance, virulence

## Abstract

The opportunistic pathogen *Pseudomonas aeruginosa* expresses a small molecular weight, cysteine-rich protein (PmtA), identified as a metallothionein (MT) protein family member. The MT family proteins have been well-characterized in eukaryotes as essential for zinc and copper homeostasis, protection against oxidative stress, and the ability to modify a variety of immune activities. Bacterial MTs share sequence homology, antioxidant chemistry, and heavy metal-binding capacity with eukaryotic MTs, however, the impact of bacterial MTs on virulence and infection have not been well-studied. In the present study, we investigated the role of PmtA in *P. aeruginosa* PAO1 using a PmtA-deficient strain (Δ*pmtA*). Here we demonstrated the virulence factor, pyocyanin, relies on the expression of PmtA. We showed that PmtA may be protective against oxidative stress, as an alternative antioxidant, glutathione, can rescue pyocyanin expression. Furthermore, the expression of *phzM*, which encodes a pyocyanin precursor enzyme, was decreased in the Δ*pmtA* mutant during early stationary phase. Upregulated *pmtA* expression was previously detected in confluent biofilms, which are essential for chronic infection, and we observed that the Δ*pmtA* mutant was disrupted for biofilm formation. As biofilms also modulate antibiotic susceptibility, we examined the Δ*pmtA* mutant susceptibility to antibiotics and found that the Δ*pmtA* mutant is more susceptible to cefepime and ciprofloxacin than the wild-type strain. Finally, we observed that the deletion of *pmtA* results in decreased virulence in a waxworm model. Taken together, our results support the conclusion that PmtA is necessary for the full virulence of *P. aeruginosa* and may represent a potential target for therapeutic intervention.

## Introduction

*Pseudomonas aeruginosa* is a non-fermenting Gram-negative, opportunistic pathogen that ranks amongst the top five hospital-associated infections. Most commonly, these bacteria are found in patients that are immunocompromised, have undergone invasive surgery and/or have underlying conditions such as diabetes (Moradali et al., 2017; Wong et al., 2021). *P. aeruginosa* infections frequently arise from associations with medical devices such as ventilators, central lines, urinary catheters and/or surgical/transplantation (Moradali et al., 2017) and have been implicated as the causative agent of a wide variety of infections (e.g., pneumonia, sepsis, keratitis, skin, bone, joint, endocarditis, and meningitis) (Gellatly and Hancock, 2013; Magalhães et al., 2016; Wong et al., 2021). *P. aeruginosa* infections associated with chronic lung disease such as cystic fibrosis disease, ventilator-associated pneumonia (VAP), and individuals with chronic obstructive pulmonary disease (COPD) are persistent and are often linked with an increase in morbidity and mortality (Moradali et al., 2017; Malhotra et al., 2019). More recently, *P. aeruginosa* has been found as a common coinfection in patients hospitalized with COVID-19 (Qu et al., 2021). A major challenge in effectively treating these infections is multidrug-resistant (MDR) strains of *P. aeruginosa*. MDR strains have been associated with increased visits to clinical settings, very poor clinical outcomes, and are considered critical on the priority list by the World Health Organization (Tacconelli et al., 2013; Shrivastava and Ramasamy, 2018; Malhotra et al., 2019). Intrinsic antimicrobial resistance genes make *P. aeruginosa* highly resistant to antibiotics. These genes include efflux pump systems, beta-lactamase production, porin alterations, and target site modifications (Chuanchuen et al., 2002; Aedekerk et al., 2005; Reygaert, 2018). Although conventional antibiotics such as cefepime and ciprofloxacin are currently used to treat *P. aeruginosa* infections, resistance rates continue to rise in the four major groups of anti-pseudomonal agents: carbapenem, aminoglycosides, cephalosporins, and fluoroquinolones (Marvig et al., 2014; Pachori et al., 2019; Gajdács et al., 2021). Therefore, without reasonable availability of new therapeutic agents, drug resistance may worsen. Recently, targeting virulence factors has emerged as a new line of action in combating MDR *P. aeruginosa* strains and has revealed 12 classes of small-molecule inhibitors and two antibodies that attack key virulence regulators (Shao et al., 2020).

To establish an infection, *P. aeruginosa* uses an array of virulence factors to counteract host defenses, cause direct damage to the host tissues and increase microbial competitiveness (Gellatly and Hancock, 2013). Some of these virulence factors involve phenazines, biofilms, exotoxins, endotoxins, proteases, siderophores, flagella, and secretion systems (Behzadi et al., 2021). Phenazines are secondary metabolites produced by *Pseudomonas* species that have been shown to increase microbial virulence (Das et al., 2016; Gonçalves and Vasconcelos, 2021). *P. aeruginosa* synthesizes a variety of these compounds, i.e., pyocyanin, phenazine-1-carboxylic acid (PCA), 1-hydroxyphenazine (1-OH-PHZ) and phenazine1-carboxamine (PCN). Construction of phenazines involves two homologous core loci biosynthetic pathways *(phzA1B1C1D1E1F1G1* and *phzA2B2C2D2E2F2G2*) along with three genes (*phzM, phzS* and *phzH*) that encode enzymes responsible for converting PCA to pyocyanin, PCN and 1-OH-PHZ. Pyocyanin is a well-characterized virulence factor produced by 95% of *P. aeruginosa* isolates (Gonçalves and Vasconcelos, 2021). Importantly, this oxidant-sensitive metabolite allows *P. aeruginosa* to manipulate the redox micro-environment during infection. Pyocyanin induces reactive oxygen species (ROS) production by host cells and blocks the host expression of catalase, an enzyme that neutralizes ROS through NADPH oxidation (Lau et al., 2004a,b). Sustained exposure to ROS results in host cell damage and a decrease in host defenses, which allows bacteria to establish a chronic infection (Gellatly and Hancock, 2013). In a murine infection model, pyocyanin-deficient mutants are more readily cleared from the lungs and less able to cause pneumonia than their wild-type counterparts (Lau et al., 2004b). Taken together, these results suggest that pyocyanin expression is essential for disease.

Another important virulence factor for *P. aeruginosa* is the ability to form biofilms. Biofilms are complex communities of microbes encapsulated in an extracellular polymeric substance (EPS) matrix that is comprised of proteins, lipids and extracellular DNA. Extracellular DNA (eDNA) has been identified as a major component of *P. aeruginosa* biofilm that is released by the lysing of a subpopulation of *P. aeruginosa* cells. In *P. aeruginosa*, eDNA release is mediated by quorum sensing (QS) signaling molecules *N*-acyl-L-homoserine lactone and Pseudomonas quinolone. Mediation of eDNA can also be affected by flagella and type IV pili mechanisms. In addition to initiating biofilm formation, eDNA can also bind to antibiotics, affording biofilm-associated cells even greater protection from these molecules. Pyocyanin is also regulated by QS and is essential for the release of eDNA, aiding in the formation of biofilms (Das et al., 2013).

**FIGURE 1:**
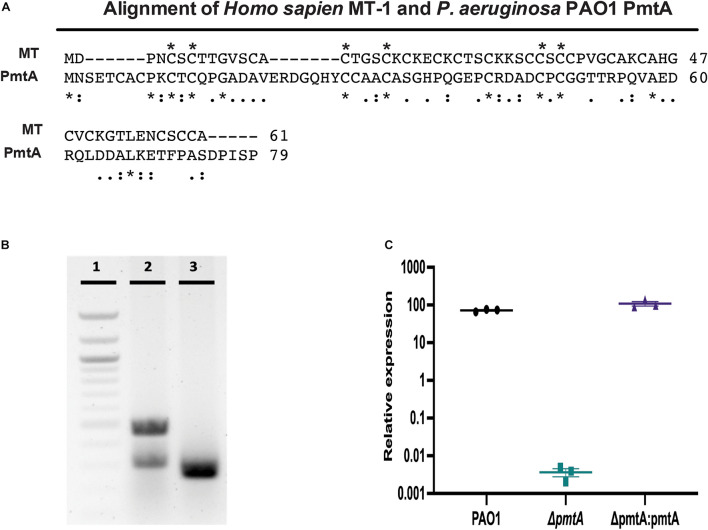
Comparison of PAO1 PmtA and Homo sapiens Metallothionein I and generation valuation of the Δ*pmtA* mutant. **(A)** MT from *P. aeruginosa* PAO1 contains 79 amino acids, 10 of which are cysteine residues. A comparison of human MT-1 (Accession: AAH29475) and PAO1 PmtA (Accession: NP_250830) using the BLAST online software reveals there is little homology between the sequences (*e* = 6), but the alignment reveals many of the fully conserved regions (*, below sequence) are cysteine residues. A (:) indicates conservation of amino acids with strongly similar properties, while (.) indicates conservation of amino acids with somewhat similar properties. Cysteine residues that are conserved between MT-1 and PmtA are indicated by (*) above the sequence. **(B)** Confirmation of clean deletion mutant was revealed by colony PCR. Lane 1, shows a 100 bp ladder; lane 2, shows wild-type PAO1 (485 bp), and lane 3, shows the Δ*pmtA* mutant (263 bp). **(C)** Overnight cultures of *P. aeruginosa* strains were grown in LB media for 24 h and total RNA was extracted from the cell pellets. Relative expression values for *pmtA* were calculated using the cycle threshold value compared to a standard curve for *pmtA*. *pmtA* expression was decreased in the Δ*pmtA* mutant. This expression was restored in the complemented strain, Δ*pmtA:pmtA*. One-way ANOVA analysis was performed (*p < 0.05; **p < 0.01; ***p < 0.001; ****p < 0.0001). The data are presented as the average of three biological replicates (± standard error of the mean) and are representative of three separate experiments.

*Pseudomonas aeruginosa* expresses a metallothionein (MT) known as PmtA (*P*. *aeruginosa* MT A) (Blindauer, 2008). Although little is known regarding the function of bacterial MTs, they do share some sequence homology, heavy metal binding characteristics, and cysteine motifs with their eukaryotic counterparts (Figure 1A and Table 1). Eukaryotic MTs are well studied in mammals for their immunomodulatory role. MTs can influence leukocyte trafficking and decrease proinflammatory mediators (Youn et al., 1995; Yin et al., 2005). Additionally, MT expression intensifies inflammatory bowel disease, alters the antibody response to antigen challenge, and anti-MT antibodies can ameliorate the severity of chronic inflammation (Lynes et al., 1993; Laukens et al., 2009; Devisscher et al., 2014).

**TABLE 1:** PmtA comparison of Heavy metal binding characteristics and cysteine motifs.

	PmtA (*Pseudomonas* species)	SmtA (*Synechococcus* species)	Mammalian MT
# of amino acids	56–79	55–56	60–68
# of cysteine residues	8–10	8–10	18–23
# of bound zinc	3–4	3–4	7–8

A classic feature of the MT protein family is an abundance of cysteine residues and the presence of free thiol groups. These allow MTs to bind divalent heavy metals and contribute to their immunomodulatory functions (Lynes et al., 1993). MTs protects the host from ROS-induced damage during oxidative stress through oxidation of thiol-groups, which may allow macrophages to produce more ROS in the presence of MTs (Borghesi and Lynes, 1996). PmtA may have similar immunomodulatory properties and potentially influence *Pseudomonas* virulence due to its similarities in structure to mammalian MT. PmtA has also been shown to be upregulated in confluent biofilms compared to planktonic cells (Hentzer et al., 2005; Waite et al., 2006).

To date, the function of PmtA in *P. aeruginosa* infections in relation to virulence is unknown. In this study, we investigated the role of PmtA in *P. aeruginosa* strain PAO1, using a clean deletion mutant (Δ*pmtA*). We also investigated the role PmtA plays in biofilm formation, pyocyanin production and antibiotic sensitivity.

## Materials and Methods

### Bacterial Strains, Plasmids, Growth Media, and Antibiotics

The bacterial strains, plasmids, and primers used in the present study are listed in Supplementary Tables S1, S2. *P. aeruginosa* strain PAO1 (Jacobs et al., 2003) was used as the wild-type strain, and all *P. aeruginosa* strains were grown at 37°C in Luria-Bertani (LB) or M9 salt liquid media or on agar-solidified plates. M9 salts (5×) contained (per liter) 30 g of Na_2_HPO_4_, 15 g of KH_2_PO_4_, 5 g of NH_4_Cl, and 2.5 g of NaCl. M9-based medium 6.25 mL of 5 × M9 salts, 625 μL of 20% glucose, 62 μL of 1 M MgSO_4_, 3.25 μL of 1 M CaCl_2_ and 24.3 mL of Nanopure water. The media were sterilized through 0.2 μm sized filters into 50-mL sterile falcon tubes and supplemented with 100 μg/mL gentamicin (GBiosciences St. Louis, MO, United States, RC-176) as needed.

### Deletion and Complementation of PmtA

An unmarked deletion mutant (Δ*pmtA*) was derived from PAO1 using a two-step allelic exchange as previously described (Hoang et al., 1998; Hmelo et al., 2015) and depicted in Supplementary Figure S1. Briefly, using the primer pairs 1_PA2140_del_F/R and 2_PA2140_del_F/R, approximately 500-bp fragments upstream and downstream of the target genes were amplified with 15–20 bp overlapping end complimentary to *Bam*HI-HF (New England BioLabs (NEB), Ipswich MA, R3136S) and *Eco*RI-HF (NEB, R3101S)-digested pEX18Gm using Q5 High-Fidelity DNA Polymerase (NEB, M0491S). The mutant gene fragments were assembled using Gibson assembly (NEB, E5510S) with double-digested pEX18Gm and transformed into One Shot PIR1 (Invitrogen, Waltham, MA, United States, C101010) chemically competent *Escherichia coli* cells. Transformants were selected on LB plates supplemented with 15 μg/mL gentamicin and screened using colony PCR with the primer pair 2_PA2140_test_F/R. Clones bearing allelic exchange vectors, in which the mutant allele was inserted, were then assessed for correctness using Sanger sequencing. Plasmids were purified, transformed into donor *E. coli* strain SM10 λ-pir and conjugated into *P. aeruginosa* PAO1 recipient cells. Transformants were selected on Vogel-Bonner minimal media containing 10 mM citrate as a carbon source to select against *E. coli*. To select against merodiploids, LB agar containing 15% (wt/vol) sucrose and no NaCl_2_ was used for counterselection. *Pseudomonas* isolation agar containing 60 μg/mL gentamicin was used to select for isolates having undergone spontaneous excision of the plasmid. Isolates that no longer could grow in the presence of gentamicin were screened for deletion of the wild-type allele using the primer pair PA2140_Tn7_F/R by colony PCR (Figure 1B) and assessed for correctness by Sanger sequencing.

A complemented deletion mutant strain (Δ*pmtA:pmtA*) was generated with pUC18R6K-mini-Tn7T-Gm (Addgene, Watertown, MA, United States, 65022) as described by Choi and Schweizer (2006). The full-length of the target gene (including a potential promoter) was amplified from *P. aeruginosa* strain PAO1 using the primer pairs PPA2140_Tn7_F/PA2140_Tn7_R. Amplification fragments were assembled using Gibson assembly with *Pst*I-HF (NEB, R3140S) and *Kpn*I-HF (NEB, R3142S)-digested pUC18R6K-mini-Tn7T-Gm and transformed into One Shot PIR1 chemically competent *E. coli* cells for directed recombination of the fragment into the plasmid. The Δ*pmtA* mutant was transformed with the suicide plasmid and selected for on LB medium containing 60 μg/mL of gentamicin. Isolates that grew in the presence of gentamicin were screened for the presence of the complementation construct using the primer pair pTn7GM_F/R by colony PCR (Supplementary Figure S4) and assessed for correctness by Sanger sequencing.

### Bacterial Growth

Overnight cultures were diluted 1:100 in fresh LB or M9 salt media without antibiotics, diluted and aliquoted into a 96-well plate (Greiner Bio-One, Monroe, NC, United States, 655101) in triplicate and then monitored for growth by measuring the optical density at 600 nm (OD_600_) in a Spectramax microplate reader (Molecular Devices, Sunnyvale, CA, United States). As *P. aeruginosa* aggregates after the first 4 h of cultivation, the strains were diluted 1:10 in LB media and vortexed before the OD_600_ was determined. For *P. aeruginosa* growth curves in LB supplemented with oxidants and metals, overnight cultures were diluted 1:100 in fresh LB medium and grown for 2 h to exponential phase. Then, 600 μL of each culture was added in triplicate to the wells of a 24-well sterile plate (Fisher Scientific, Waltham, MA, United States, Cat#353047) together with 10 mM hydrogen peroxide, 100 μM CdCl_2_, 200 μM ZnCl_2_, or medium. The strains were cultured for 10–12 h with intermittent shaking at 37°C, after which growth was calculated by dividing the average OD_600_ value of triplicate cultures grown with oxidant by that obtained for cultures grown without oxidant. For M9 salt medium, overnight cultures were subcultured to an OD_600_ of 0.08, and 600 μL was added to the wells of a 24-well sterile plate (Falcon, Cat#353047) in triplicate and mixed with 400 μL of M9 salt medium alone or supplemented with oxidants or metals (200 μg/mL transferrin, 150 μM ethylenediaminediacetic acid (EDDA), 50 μM FeCl_2_, 10 mM hydrogen peroxide, 200 μM CdCl_2_, 100 μM ZnCl_2_). The strains were cultured for 18 h with intermittent shaking at 37°C. To measure pyocyanin production, single colonies of the *P. aeruginosa* strains were grown overnight in 5 mL of LB medium with the appropriate antibiotics at 37°C with vigorous shaking. These cultures were then diluted at 1:100 in fresh LB media without antibiotics at a dilution of 1:100 and grown at 37°C with vigorous shaking. Pyocyanin concentrations were determined as previously described (Frank and Demoss, 1959) with the exception that 1 mL of each culture was centrifuged for 1 min at 20,500 × *g*. The supernatants were transferred to new microcentrifuge tubes, vortexed vigorously and then transferred to a 96-well plate in triplicate to measure the absorbance at 691 nm, with measurements taken at selected time points over 30 h of cultivation. The OD_600_ values of the cultures were also monitored, and data was plotted as OD_600_ vs. Abs_691_.

### Pyocyanin Extraction

*Pseudomonas aeruginosa* strains were grown for 30 h in triplicate, and 1 mL of cell-free supernatants were obtained. Pyocyanin was extracted as previously described with minor modifications (Frank and Demoss, 1959; Essar et al., 1990; Zaborin et al., 2012). In brief, 500 μL of chloroform was added to 1 mL of supernatant from each strain. The mixture was then vigorously vortexed for 2 min as the chloroform turned blue green, after which the tubes were centrifuged for 10 min at 3000 × *g* and 4°C. Hydrochloric acid (150 μL, 0.2 M) was added to the partially extracted blue pyocyanin and vortexed vigorously for 2 min. The top blue layer was acidified to a pink color and samples were centrifuged for 2 min at 3000 × *g* and 4°C. The absorbance of completely extracted pyocyanin at the pink stage was measured at 520 nm. Measurements were normalized to initial cell density OD_600_.

### RT-qPCR

*Pseudomonas aeruginosa* strains were grown overnight in LB medium to stationary phase and a subculture was used to collect total RNA extracted from the cell pellets using QIAGEN RNeasy kit (74104) according to the manufacturer’s recommendations at 4, 10, and 24 h. RNA was converted to cDNA using the TaqMan Reverse Transcription kit (Invitrogen, N8080234). Gene-specific primers to *pmtA* and the housekeeping ribosomal gene (RNA polymerase, subunit alpha) were designed and are listed in Supplementary Table S2. qPCR was performed on an Applied Biosystems 7300 instrument using iTaq Universal SYBR green (Bio-Rad, Hercules, CA, United States, 1725121). All qPCR experiments were performed with at least three biological replicates, each tested in triplicate for each time point. The absolute gene expression for *pmtA* was calculated using the cycle threshold value compared to a standard curve of *pmtA* using pGEX-6p-pmtA. The qPCR assay efficiency was verified using a five-fold dilution standard curve in triplicate. The *R*^2^ of the standard curve was 0.99 and efficiency was 100%. qPCR was performed on an Applied Biosystems 7300 instrument using iTaq Universal SYBR green (Bio-Rad, 1725121). All qPCR experiments were performed with at least three biological replicates, each tested in quadruplicate for each timepoint. The relative gene expression for *phzH* and *phzM* was calculated using the cycle threshold value compared to the ribosomal RNA gene as the internal reference standard. Fold changes were expressed as 2^–ΔΔ*Ct*^ values. No template and wild-type PAO1 were used negative and positive control, respectively.

### Biofilm Formation Assay

The wild-type, Δ*pmtA*, and Δ*pmtA:pmtA* PAO1 strains were inoculated into 5 mL of LB and vigorously shaken at an angle in 14-mL Falcon tubes overnight. The medium was removed the next day, and the splash zone was stained for 15 min with 0.1% crystal violet (Fisher Science, Waltham, MA, United States, S25275A). Subsequently, the tubes were gently rinsed with water (2×) and inspected for the presence of a biofilm. Biofilm formation was also quantified using a previously described microtiter plate assay with minor adaptations (O’Toole, 2010). In brief, overnight cultures were diluted at 1:10 in fresh LB medium and grown for 24 h at 37°C without shaking. Growth yields were determined by measuring the absorbance at 595 nm. The medium was removed, and the biofilms were fixed to the sides of the microtiter wells with 90% methanol before being stained with 0.1% crystal violet (Fisher Science, Waltham, MA, United States, S25275A). The crystal violet was solubilized in ethanol:acetone (4:1, v/v) and measured at 595 nm using an ethanol solution as blank. *P. aeruginosa* PAO1 was used as a positive control, with three biological replicates and eight technical replicates performed for each strain. The relative biofilm formation (RBF) for each well was determined by dividing the crystal violet absorbance reading by the growth yield absorbance reading.

### Acyl Homoserine Lactone-Dependent Quorum Sensing Assay

The reporter strain *E. coli* MG4 pKDT17 (Addgene, Watertown, MA, United States, 27503) was used in crossfeeding assays with the wild-type, Δ*pmtA* or Δ*pmtA:pmtA* PAO1 strains and *E. coli* MC1061 (negative control) to assess acyl homoserine lactone (AHL) production as previously described (Pearson et al., 1995; Van Laar et al., 2018). In brief, wild-type, Δ*pmtA*, and Δ*pmtA:pmtA* PAO1 strains and *E. coli* MG4 were grown overnight. Subsequently, each PAO1 strain was streaked onto a MacConkey agar plate 0.75 cm from a streak of *E. coli* MG4 and incubated overnight at 37°C. The next day, the plates were examined for color change, where pink or purple indicated the presence of AHLs and white indicated their absence.

### Minimum Inhibitory Concentration Determination Using *E*-Test

The susceptibility of *P. aeruginosa* strains to ciprofloxacin, cefepime and tetracycline, was assessed using Liofilchem^®^ MTS^TM^ *E*-tests (Fisher Scientific, Waltham, MA, United States, 22-777-785, 22-777-917, 22-777-902) following the manufacturer’s protocol. In short, fresh *P. aeruginosa* colonies were resuspended in 1 mL of LB, and a cotton swab was then used to spread the inoculated LB over LB agar plates. The plates were allowed to dry, and then the *E*-test strip was then placed onto the inculcated agar, with each strain assayed in triplicate. The plates were incubated overnight at 37°C, and the minimum inhibitory concentration (MIC) was read the next day.

### *In vivo Galleria mellonella* Larvae Model of Infection

Healthy *Galleria mellonella* larvae (waxworms) were purchased from Vanderhorst Wholesale, Inc. (St Marys, OH, United States) and briefly stored at 4°C until use. Injections were performed as described in Seed and Dennis (2008) with a few adaptations. Cultures of *P. aeruginosa* strains were grown overnight, and the ability of 10^3^CFU/mL of each *P. aeruginosa* to cause melanization was assessed. Worms were randomly separated into groups and injected into the hindmost left proleg with 5 μL of sterile 10 mM MgSO_4_ or 5 μL of 10^3^CFU/mL of the wild-type Δ*pmtA* or Δ*pmtA:pmtA* PAO1 strains using a 10-μL Hamilton syringe (Hamilton Company, Reno, NV, United States) with a beveled needle. The inoculum (10^3^CFU/mL) was verified by plating, and the injected worms were incubated at 37°C 48 h, with survival assessed at 20, 24, 28, and 48 h post injection (Tsai et al., 2016).

## Results

### The *pmtA* Clean Deletion Mutant Has no Detectable Metallothionein Production

Despite being highly conserved, little is known regarding the physiological function and expression of *pmtA* during the life cycle of *P. aeruginosa*. We constructed a clean deletion mutation of Δ*pmtA* in wild-type *P. aeruginosa* PAO1 using a two-step allelic exchange method as well as a complemented strain (Δ*pmtA:pmtA*) by cloning *pmtA* in a Tn*7* and conjugating it into Δ*pmtA.* We verified the genetic change in the Δ*pmtA* strain using colony PCR and Sanger sequencing (Figure 1B, Supplementary Figure S2 and data not shown). The colony PCR shows a band shift when comparing the products from the PAO1 (485 bp) and Δ*pmtA* (263 bp) strains, indicating success in generation of a clean mutation. RT-qPCR was also used to assess *pmtA* transcription levels in the wild-type, Δ*pmtA* and Δ*pmtA:pmtA* PAO1 strains. The wild-type and Δ*pmtA:pmtA* strains had approximately equal amounts of transcript, while little to no *pmtA* transcript was present in Δ*pmtA* mutant cultures at 4, 10, and 24 h (Figure 1C). These results also suggest that *pmtA* is expressed throughout stationary phases in PAO1.

### The Δ*pmtA*, and Δ*pmtA:pmtA* PAO1 Strains Have Similar Growth Kinetics

**FIGURE 2:**
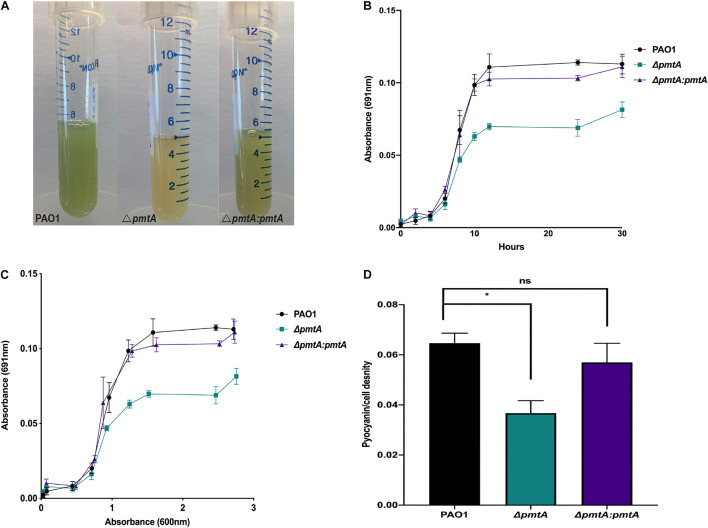
*P. aeruginosa* PAO1 strain Δ*pmtA* produces less pyocyanin than wild-type PAO1. **(A)** *P. aeruginosa* strains were each grown overnight in 5 mL of LB medium at 37°C for 24 h with vigorous shaking. The cultures were observed for color change and the Δ*pmtA* mutant had a yellow appearance compared to wild-type PAO1. **(B)** Pyocyanin levels were measured at 0, 2, 4, 6, 8, 10, 12, 24, and 30 h using 1 mL of the culture and centrifuged for 1 min at 20,500 × *g*. The supernatant was then placed in a 96 well plate in triplicate and the absorbance at 691 nm was measured. **(C)** The data was plotted as OD_600_ vs. OD_691_. **(D)** *P. aeruginosa* strains were grown for 24 h, and cell-free supernatants were obtained. Pyocyanin was extracted as previously described, with minor modifications (Essar et al., 1990; Zaborin et al., 2012). Chloroform was added to the supernatant from each strain, forming a blue bottom layer. Subsequently, hydrochloric acid (1.5 mL, 0.2 M) was added to the layer altering the pH to 2 and pink in color. The absorbance of completely extracted pyocyanin was measured at OD_520_ and the pyocyanin/cell density was calculated by dividing the pyocyanin at OD_520_ by growth measured at OD_600_. One-way ANOVA analysis was performed (*p < 0.05; **p < 0.01; ***p < 0.001; ****p < 0.0001. The data are presented as the average of three biological replicates (± standard error of the mean) and are representative of three separate experiments.

The growth kinetics of the wild-type, Δ*pmtA*, and Δ*pmtA:pmtA* PAO1 strains were examined in both LB and M9 salt media. While no significant differences in the growth rates were detected between the strains (Supplementary Figures S3A–C), the wild-type and Δ*pmtA:pmtA* strains turned the media green after 10 h of growth, while the Δ*pmtA* culture media remained yellow (Figure 2A). This color difference persisted to 30 h of growth and is likely due to a difference in the production of pyocyanin, which is known to be secreted by PAO1 and imparts a distinct bluish-green color to media (Hassan and Fridovich, 1980).

### The Δ*pmtA* Mutant Produces Less Pyocyanin Than Wild-Type PAO1

We investigated the ability of the Δ*pmtA* mutant to produce pyocyanin by determining the absorbance values of cell-free culture supernatants at 691 nm in a time course assay (Rada and Leto, 2013). Pyocyanin levels were decreased in the culture supernatants of the Δ*pmtA* strain compared to the wild-type and Δ*pmtA:pmtA* strains starting at 10 h and remained lower than those of the wild-type strain throughout the assay (Figure 2B). The highest levels of pyocyanin expression by PAO1 are observed during stationary phase growth (Rada and Leto, 2013). We compared cell growth to pyocyanin levels (691/600 nm ratio) for the various PAO1 strains, and the normalized pyocyanin levels were consistently higher in the wild-type and Δ*pmtA:pmtA* than in the Δ*pmtA* mutant (Figure 2C). QS molecules, phenazine-producing genes (*phzA-G*), and the gene *phzM* are involved in pyocyanin production at early stationary phase. Wild-type PAO1 starts to produce detectable pyocyanin at 10 h (Das et al., 2016; Figures 2B,C). These results indicate that *pmtA* is necessary for optimum pyocyanin levels in *P. aeruginosa* and that the restoration of *pmtA* expression is sufficient for re-establishing pyocyanin to normal levels.

Pyocyanin appears as different colors in media depending on the pH and oxidation status of the culture (Rada and Leto, 2013). Highly oxygenated cultures are green due to the accumulation of oxidized pyocyanin, whereas those that are not well oxygenated appear yellow due to reduced pyocyanin (Rada and Leto, 2013). To ensure that the 691/600 nm ratio of wild-type PAO1 was not higher than that of the Δ*pmtA* mutant due to a difference in the pH or oxidation status, pyocyanin was extracted with chloroform/HCl after 24 h of growth, and the absorption readings of the supernatants were analyzed as previously described (Rada and Leto, 2013). Wild-type PAO1 extracts were significantly higher than the Δ*pmtA* mutant extracts, while extracts were restored to wild-type levels in the complemented mutant Δ*pmtA:pmtA* (Figure 2D). These results indicate that wild-type PAO1 produces more pyocyanin than the Δ*pmtA* mutant and the difference in the 691 nm readings is not due to a difference in the oxidation state of pyocyanin or the pH of the culture.

### Exogenous Addition of the Antioxidant Glutathione Restores Pyocyanin Production to Wild-Type Levels in the Δ*pmtA* Mutant

**FIGURE 3:**
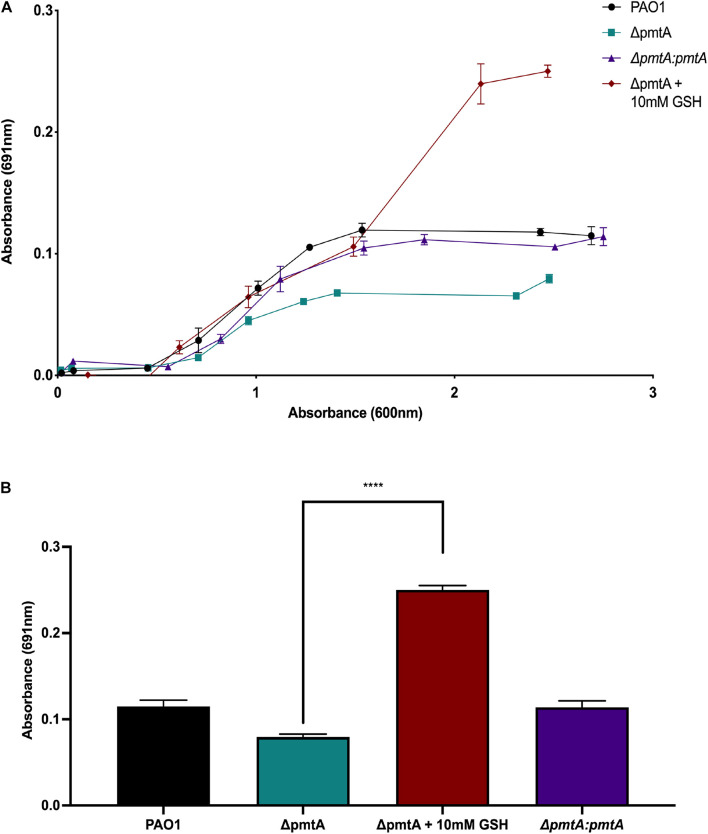
Exogenous addition of the antioxidant glutathione restores pyocyanin production in the *P. aeruginosa* Δ*pmtA* mutant. **(A)** The wild-type, Δ*pmtA*, Δ*pmtA:pmtA* and Δ*pmtA* supplemented with 10 mM glutathione (GSH) PAO1 strains were cultured for 30 h at 37°C with vigorous shaking. Over the 30-h, the growth of the bacteria (OD_600_) was compared to the production of pyocyanin in media (OD_691_). The wild-type and Δ*pmtA:pmtA* PAO1 strains produced more pyocyanin when compared to the Δ*pmtA* mutant. Pyocyanin production of the Δ*pmtA* mutant was restored to near wild-type levels starting at 8 h of growth. **(B)** The wild-type, Δ*pmtA*, Δ*pmtA:pmtA*,Δ*pmtA* and Δ*pmtA* supplemented with 10 mM glutathione (GSH) PAO1 strains were cultured in LB medium for 30 h at 37°C with vigorous shaking. At 30 h, pyocyanin was measured in the media (OD_691_). The wild-type and Δ*pmtA:pmtA* strains produced more pyocyanin when compared to the Δ*pmtA* mutant. The pyocyanin production of the Δ*pmtA* mutant was significantly increased with the addition of GSH. An unpaired *t*-test analysis was performed (*p < 0.05, **p < 0.01, ***p < 0.001, ****p < 0.0001). The data are presented as the average of three biological replicates (± standard error of the mean) and are representative of three separate experiments.

Because the Δ*pmtA* mutant produces less pyocyanin, we hypothesize that this strain also has a reduction in antioxidant capacity. To test this hypothesis, the alternative antioxidant glutathione (GSH) was added to the growth media, and changes in pyocyanin production were monitored. The absorbance values at 691 and 600 nm were measured at 0, 2, 4, 6, 8, 10, 12, 24, and 30 h of growth and the 691/600 nm ratios were calculated (Figure 3A). The addition of biological levels of GSH restored pyocyanin to wild-type levels, indicating that PmtA alters the redox environment and influences pyocyanin production (Figure 3B; Meister and Anderson, 1983).

### PmtA Plays an Essential Role in Phenazine Production

**FIGURE 4:**
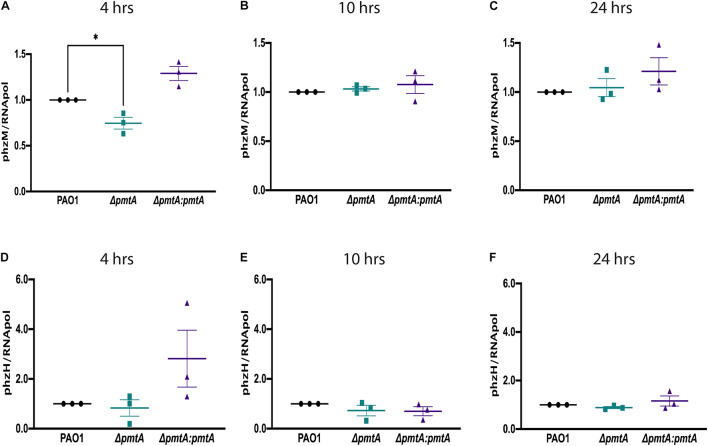
PmtA is required for the early expression of the phenazine pigment precursor 5-methylphenazine-1-carboxylic acid betaine. Overnight cultures of *P. aeruginosa* strains were grown in LB medium for 4, 10, and 24 h, and total RNA was extracted from the cell pellets. RNA was converted to cDNA using a TaqMan Reverse Transcription kit. qPCR was performed on an ABI7300 instrument using SYBR green fluorescent dye and ROX as a detector. The relative gene expression values for *phzM* **(A–C)** *and phzH* **(D–F)** were calculated using the cycle threshold value compared to the RNA polymerase, subunit alpha. **(A)** The expression of *phzM* at 4 h decreased in the Δ*pmtA* mutant and was restored in the complemented mutant Δ*pmtA*:*pmtA*. CT (cycle threshold) values were used to calculate RQ (relative quantitation) values for *phzH* and *phzM* at each time point and normalized to the housekeeping gene RNA polymerase. An unpaired *t*-test analysis (*p < 0.05, **p < 0.01, ***p < 0.001, ****p < 0.0001). The data are presented as the average of three biological replicates (± standard error of the mean) and are representative of three separate experiments.

The *phzA1* and *phzA2* gene clusters encode enzymes required for phenazine synthesis to produce phenazine-1-carboxylic acid. PhzH converts phenazine-1-carboxylic acid to phenazine-1-carboxamide, while PhzM converts phenazine-1-carboxylic acid to 5-methylphenazine-1-carboxylic acid betaine, which is later converted to pyocyanin by PhzS (Mavrodi et al., 2001). To elucidate the mechanism by which PmtA affects pyocyanin biosynthesis, we assessed the expression of *phzH* and *phzM* using real-time PCR (Figure 4). The expression of *phzM* but not *phzH* was decreased in the Δ*pmtA* mutant (Figure 4A), and *phzM* expression was restored in the complemented mutant Δ*pmtA:pmtA* at 4 h. However, no differences were observed between the wild-type, Δ*pmtA* and Δ*pmtA:pmtA* strains at the later time points (10 and 24 h). These results suggest that PmtA biosynthesis is required for the early expression of *phzM* but not *phzH via* unknown mechanisms and that the expression levels of this gene contribute to the overall production of the pyocyanin precursor 5-methylphenazine-1-carboxylic acid betaine in *P. aeruginosa*.

### PmtA Is Essential for Biofilm Formation

**FIGURE 5:**
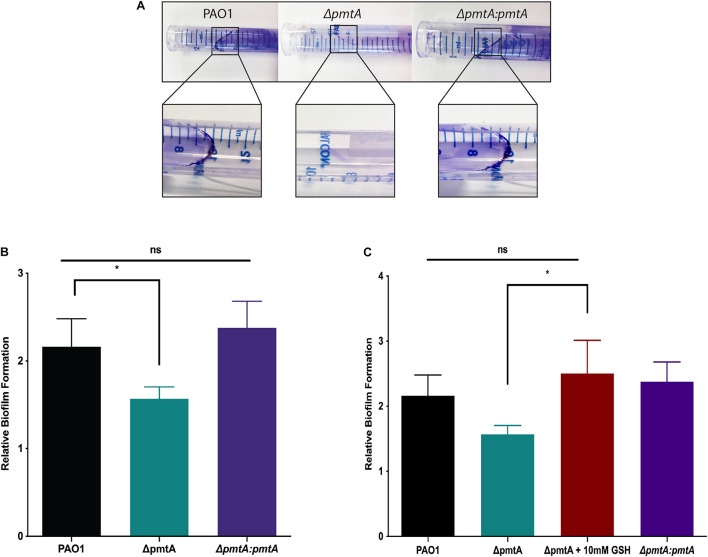
PmtA is required for biofilm formation and the addition of antioxidant glutathione restores biofilm formation in *P. aeruginosa*Δ*pmtA* mutant. **(A)** Cultures of *P. aeruginosa* strains were grown overnight in LB medium and stained with crystal violet. Less crystal violet was observed in the Δ*pmtA* mutant when compared to wild-type and Δ*pmtA*:*pmtA* PAO1 strains. **(B)** Cultures of *P. aeruginosa* strains were grown overnight in LB media, diluted 1:20 in fresh LB medium and grown to 0.5 OD_600_. After 24 h of growth, growth yield was measured at OD_600_. Biofilms were stained as previously described with minor modifications (O’Toole, 2010) and crystal violet stain was measured at OD_595_. The OD reading after the addition of crystal violet was divided by the growth yield OD to calculate the relative biofilm formation. **(C)** GSH at 10 mM was added to the LB media in the 96 well plate assay to determine if an antioxidant would rescue the biofilm formation defect in the Δ*pmtA* mutant. GSH restored the biofilm formation of the Δ*pmtA* mutant in the microtiter plate assay. An unpaired *t*-test analysis was performed (*p < 0.05; **p < 0.01; ***p < 0.001; ****p < 0.0001). The data are presented as the average of three biological replicates (± standard error of the mean) and are representative of three separate experiments.

As upregulated PmtA expression has been observed in confluent *P. aeruginosa* biofilms (Hentzer et al., 2005; Waite et al., 2006), we investigated the role of PmtA in biofilm formation. First, we assessed biofilm formation by the wild-type, Δ*pmtA*, and Δ*pmtA:pmtA* PAO1 strains by examining the splash zones of overnight cultures stained with crystal violet. As shown in Figure 5A, distinct crystal violet-stained biofilms were formed by the wild-type and Δ*pmtA:pmtA* strains but not the Δ*pmtA* mutant. Additionally, a crystal violet microtiter plate biofilm assay was used to quantitate the biofilm formation defect presented by the Δ*pmtA* strain. Interestingly, the Δ*pmtA* mutant showed a 28% decrease in biofilm formation compared to the wild-type and Δ*pmtA:pmtA* PAO1 strains (Figure 5B). These results indicate that the lack of PmtA contributed to decreased biofilm production by the Δ*pmtA* mutant and that the restoration of PmtA production resulted in the reestablishment of biofilm formation. Incidentally, the addition of the antioxidant GSH restored biofilm formation in the Δ*pmtA* mutant, confirming that redox plays a role in *P. aeruginosa* biofilm formation (Figure 5C).

### PmtA Is Not Essential for Managing Oxidative or Metal Stress

Low-molecular-weight thiols are essential for detoxifying oxidative stressors and ameliorating heavy metal stress (Van Laar et al., 2018; Ulrich and Jakob, 2019). For example, the low-molecular-weight thiol OspR can act as an oxidative stress sensor in *P. aeruginosa*. MTs can actively scavenge reactive oxygen and nitrogen species due to their high abundance of cysteine residues (Ruttkay-Nedecky et al., 2013). In a previous study, *pmtA* expression was shown to be upregulated upon 10 mM H_2_O_2_ stimulation for 15 min compared to untreated controls (Goldová et al., 2011). Therefore, we assessed whether the lack of PmtA contributes to increased sensitivity to various oxidants and heavy metals. First, we characterized the growth of the Δ*pmtA* mutant compared to the wild-type and Δ*pmtA*:*pmtA* PAO1 strains in the presence of 10 mM H_2_O_2_. All three strains grew to similar levels in both LB and M9 salt media supplemented with 10 mM H_2_O_2_ (Supplementary Figure S4). These results indicate that PmtA alone does not detoxify peroxides and that other factors may contribute to this process.

Zinc functions as a cofactor for many essential enzymes in all organisms. However, as excess zinc can be toxic, bacteria maintain intracellular zinc concentrations at low levels (Blindauer, 2015). Cadmium is toxic to most organisms and generates oxidative stress (Maret and Moulis, 2013). MTs play a role in zinc and copper homeostasis as well as cadmium detoxification (Blindauer, 2011). Cyanobacterial MTs are encoded within defined operons that include a zinc-dependent repressor (SmtB) and an operator promoter region in the intergenic region (Morby et al., 1993; Blindauer, 2008, 2011). However, *Pseudomonas* MT-harboring operons appear to lack such features and encode proteins of unknown functions (Figure 1A and Table 1). In the present study, we investigated the role of PmtA in heavy metal resistance by culturing the wild-type, Δ*pmtA*, and Δ*pmtA*:*pmtA* PAO1 strains in LB and in M9 salt media supplemented with zinc or cadmium and assessing their viability over time (Supplementary Figure S5). All strains grew to similar levels in both media types, with no differences observed between the strains exposed to inhibitory concentrations of ZnCl_2_ (200 μM) (Supplementary Figures S5A,B) or CdCl_2_ (100 μM) (Supplementary Figures S5C,D). In addition, no differences were observed between the strains when cultured for 6 h before exposure (data not shown). The similar growth pattern of the strains under zinc or cadmium excess suggests that PmtA is not crucial for metal resistance.

As iron uptake is also associated with pyocyanin production, we examined the ability of the wild-type, Δ*pmtA*, and Δ*pmtA*:*pmtA* strains to grow iron chelator EDDA. All strains had a reduced growth rate when compared to growth in M9 salt media indicating the iron limiting conditions. FeCl_2_ and transferrin restored the growth of all the stains showing that iron utilization is not affected by the loss of PmtA (Supplementary Figure S6).

### PmtA Does Not Inhibit Acyl Homoserine Lactone (AHL)-Mediated Quorum Sensing

**FIGURE 6:**
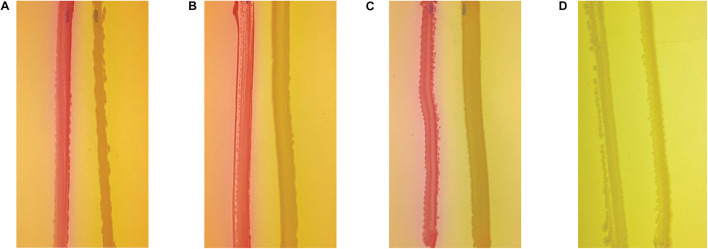
PmtA does not affect acyl homoserine lactones levels. Wild-type **(A)**, Δ*pmtA* **(B)**, and Δ*pmtA*:*pmtA* **(C)**. PAO1 **(D)** *E. coli* MC1061 strains were streaked out on MacConkey agar plates 0.75 cm apart from *E. coli* strain MG4. In the presence of AHL, lasR initiates the transcription of LasB-LacZ fusion protein resulting in beta-galactosidase in MG4, this causes colonies to change color from white to pink. All three strains caused the color change to pink suggesting that QS mediated by AHL is not inhibited by the absence of PmtA.

Quorum sensing has been shown to mediate many processes in *P. aeruginosa*, including pyocyanin production and biofilm formation. Therefore, we assessed whether AHL-dependent QS is altered in the Δ*pmtA* mutant using the reporter strain *E. coli* MG4 (pKDT17). In this strain, LasR transcribes a *lasB-lacZ* gene fusion in the presence of AHLs, and the resulting β-galactosidase fusion protein generates a color change in colonies from white to pink/purple when streaked on MacConkey agar. The wild-type, Δ*pmtA*, and Δ*pmtA*:*pmtA* PAO1 strains all induced a color change when streaked on MacConkey agar, indicating that AHL was produced and that PmtA does not mediate AHL-dependent QS (Figures 6A–C). As a negative control, strain *E. coli* MC1061 was also assessed, no color change was observed (Figure 6D).

### PmtA Promotes Antibiotic Resistance in PAO1

**FIGURE 7:**
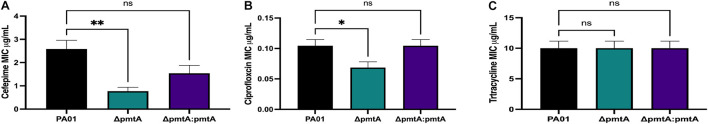
The effects of PmtA on the Minimum Inhibitory Concentration (MIC) of cefepime, ciprofloxacin and tetracycline. Single colonies from the wild-type, Δ*pmtA*, and Δ*pmtA:pmtA* PAO1 strains were each inoculated into 1 mL of LB. A sterile cotton swab was used to spread the culture over a LB plate. Liofilchem^®^ MTS *E*-tests were placed separately onto the plates after inoculation for cefepime **(A)**, ciprofloxacin **(B)** and tetracycline **(C)**. When compared to wild-type and Δ*pmtA*:*pmtA* PAO1 strains, the Δ*pmtA* mutant was significantly more susceptible to cefepime and ciprofloxacin. One-way ANOVA analysis was performed (*p < 0.05; **p < 0.01; ***p < 0.001; ****p < 0.0001. The data are presented as the average of three biological replicates (± standard error of the mean) and are representative of three separate experiments.

Weakened resistance to antibiotics is a classic biofilm defect characteristic. Therefore, we examined the sensitivity of the Δ*pmtA* mutant to tetracycline, to which *P. aeruginosa* is intrinsically resistant, and two of the conventional antibiotics used to treat CF patients infected with *P. aeruginosa*, cefepime and ciprofloxacin. Using test strips, we quantitatively assessed the MIC of the antibiotics on the wild-type, Δ*pmtA*, and Δ*pmtA*:*pmtA* strains (Figure 7). Compared to the wild-type and Δ*pmtA*:*pmtA* PAO1 strains, the Δ*pmtA* mutant exhibited significantly lower MICs for cefepime and ciprofloxacin, indicating that PmtA promotes resistance to these antibiotics. However, no differences were observed among strains exposed to tetracycline, suggesting that PmtA does not affect *P. aeruginosa* growth in the presence of this antibiotic, to which it is naturally resistant.

### PmtA Expression Influences *Pseudomonas aeruginosa* Virulence *in vivo*

**FIGURE 8:**
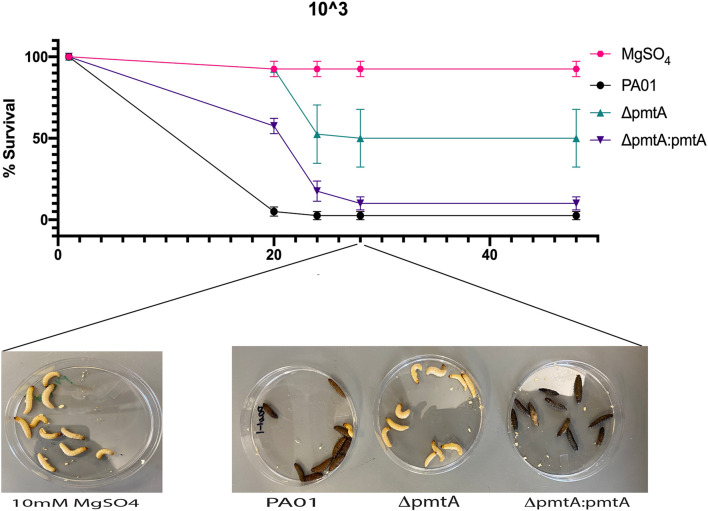
Wild-type PAO1 is more lethal than the Δ*pmtA* mutant in a *G. mellonella* model. *G. mellonella* were injected with 5 μL of 10^3^/mL of either *P. aeruginosa* wild-type, Δ*pmtA* and Δ*pmtA*:*pmtA* PAO1 strains or 10 mM of MgSO_4_ (control). *G. mellonella* survival was observed for 48 h for all four groups, and percent survival was calculated. Δ*pmtA* had a higher survival percentage than PAO1 and Δ*pmtA*:*pmtA.* The data are presented as the mean and standard deviation of the mean of three independent experiments (*n* = 10/group).

The correlation between PmtA expression and the crucial virulence factors pyocyanin and biofilm formation indicates that PmtA may contribute to the overall virulence of *P. aeruginosa*. To evaluate the virulence of the Δ*pmtA* mutant, we used *G. mellonella* larvae, a model system used to test the virulence of infectious bacteria and the innate immune response to cope with infection (Hoffmann, 1995; Mulcahy et al., 2011; Haller et al., 2014). Larvae were injected with 5 μL of diluted cultures containing 10^3^ cells of the wild-type. Δ*pmtA*, or Δ*pmtA:pmtA* strains. Survival of the larvae was evaluated 20, 24, 28 and 48 h after injection (Figure 8). At 20 h, 100% of the larvae injected with the Δ*pmtA* mutant survived compared to 10% of those injected with the wild-type strain, a 90% difference in survival rates. Compared to the Δ*pmtA* mutant, the larvae injected with the complemented strain showed a 42% decrease in survival at 20 h. At 24 h, the percent survival of larvae injected with the Δ*pmtA* mutant decreased to 52.5%, while those injected with Δ*pmtA:pmtA* and wild-type was less than 20%. These data indicate a role for PmtA in the virulence of *P. aeruginosa.*

## Discussion

Pyocyanin and biofilm formation are powerful virulence factors for *P. aeruginosa*, especially in invasive lung infections. Pyocyanin is easily diffusible through host membranes and can induce a variety of harmful cellular effects including neutrophil apoptosis, alter the expression of a number of cytokines and change the redox environment leading to the persistence of harmful oxidants produced by host polymorphonuclear leukocytes (PMNs) and macrophages (Usher et al., 2002; Managò et al., 2015; Hall et al., 2016). Here, we show for the first time that a bacterial MT, PmtA, can alter the amount of pyocyanin produced by *P. aeruginosa*, thereby influencing biofilm formation.

Pyocyanin can influence biofilm formation in two different ways. First, it can directly accept electrons from NADH or NADPH during aerobic respiration. This process passes electrons to the final electron donor to generate reactive oxygen species (e.g., superoxide and hydrogen peroxide) (Gonçalves and Vasconcelos, 2021). The oxidative stress caused by ROS production has been shown to cause a portion of the *P. aeruginosa* cell population to undergo autolysis, releasing eDNA, suggesting that pyocyanin can mediate eDNA production and help promote biofilm formation (Okshevsky and Meyer, 2015; Das et al., 2016; Malhotra et al., 2019). Pyocyanin has also been shown to influence biofilm formation by directly interacting with eDNA. This binding can mediate aggregation and cell–cell interaction within biofilms, stabilizing the biofilm structure (Das et al., 2013). eDNA is an essential component of the extracellular polymeric substance produced by *P. aeruginosa* and has been implicated in early attachment/biofilm formation and resistance to antimicrobials (Malhotra et al., 2019). The Δ*pmtA* mutant exhibited a defect in biofilm formation and/or stabilization, which is likely due to less pyocyanin availability. Interestingly, GSH restored both the pyocyanin levels and the biofilm defect in the Δ*pmtA* strain. GSH neutralizes oxidative stress, mediated by pyocyanin, and *P. aeruginosa gsh* mutants produce less pyocyanin (Van Laar et al., 2018). Together with previous data indicating that PmtA may be upregulated after oxidant exposure, these results suggest a role for PmtA in relieving oxidative stress. Alternatively, PmtA may interact with *P. aeruginosa*-derived GSH to promote pyocyanin levels. Future studies will focus on investigating potential PmtA interactions.

We also observed a decrease in the expression of *phzM*, a gene that encodes for methyl-transferase. This enzyme helps convert phenazine-1-carboxylic acid to 5-methylphenazine-1-carboxylic acid betaine allowing for the synthesis of pyocyanin. The low transcript levels of *phzM* detected in the Δ*pmtA* mutant compared to the wild-type strain correspond with the pyocyanin production phenotypes observed for these strains. Interestingly, PhzM expression is QS mediated, and QS has been shown to enhance the stress response in *P. aeruginosa*, allowing for less social cheating to occur in the population (García-Contreras et al., 2014; Chen et al., 2019). Although we did not observe a defect in AHL production within the Δ*pmtA* mutant, this does not rule out that PmtA could interact with one of the four QS systems. The mechanism by which PmtA influences *phzM* expression in early stationary cultures remains to be understood.

Low molecular-weight thiols are essential to the detoxification of oxidative stressors, and our data suggests that PmtA may play a role in relieving oxidative stress. Interestingly, the Δ*pmtA* mutant showed no growth defect when cultured in the presence of inhibitory levels of oxidative or heavy metal stressors, indicating that PmtA is not essential under these conditions. A potential reason for this lack of sensitivity may be the upregulation of yet to be identified proteins and enzymes involved in protection against these stresses that could compensate for the reduction in PmtA expression. In mycobacterial thiol mutants, defective for mycothiol and ergothioneine production, organic hydroperoxide resistance protein (Ohr) levels are upregulated to promote oxidative stress protection (Ta et al., 2011; Emani et al., 2013; Singh et al., 2016). Like mycobacteria, the absence of PmtA in the Δ*pmtA* strain may lead to upregulation of essential proteins for protection against oxidative stress. However, if this were the case, the phenotypic restoration that occurred upon the addition of GSH may not have been observed.

Alternatively, as *P. aeruginosa* has many systems dedicated to the regulation of oxidative and heavy metal stress (i.e., a catalase, siderophores, efflux pumps and metal reduction), these systems may compensate for the lack of PmtA in the Δ*pmtA* mutant, resulting in no defect being observed under the assayed growth conditions. Previous studies have shown similar results where the absence of GSH had no impact on sensitivity to hydrogen peroxide or the organic peroxide cumene hydroperoxide (CHP) (Van Laar et al., 2018). Some cyanobacteria also regulate MT production by SmtB, a zinc-dependent repressor. The PmtA operon does not encode metal-responsive regulators (Morby et al., 1993; Blindauer, 2008). Instead, the predicted operon includes two genes, one with unknown function and putatively short-chain dehydrogenase (Mao et al., 2009). In addition, Habjanič et al. (2020) noted that PflQ2 MT expression was elevated during stationary phase with or without the addition of metals in *P. fluorescens* Q2-87. The addition of metals at exponential phase did not induce transcription of MT, and increasing metal concentrations resulted in a decline in growth. At both exponential and stationary phases, we also did not observe any difference in growth among the assayed strains, supporting the conclusion that PmtA is not regulated by heavy metals.

Antibiotic resistance is a worldwide health crisis and the WHO has recently added carbapenem-resistant *P. aeruginosa* to the list of pathogens for which there is a critical need for new antibiotic treatments (Pang et al., 2019). Prolonged persistence of oxidants at low levels may be advantageous for antibiotic protection in bacteria. *P. aeruginosa* expresses efflux pumps that serve to expel antibiotics from the bacterial cell and thus protect the cell. An oxidant-sensitive negative regulator, MexR, controls the expression of this efflux pump (Poole et al., 1996). Oxidants cause a conformational change in MexR, which drives efflux pump transcription (Chen et al., 2008). Therefore, a decrease in extracellular oxidant production could limit the effective expression of the efflux pump and make *P. aeruginosa* more susceptible to antibiotics. In the present study, we showed that the Δ*pmtA* mutant is more susceptible to two of the conventional antibiotics used for treatments of *P. aeruginosa* in cystic fibrosis patients. These results suggest that PmtA expression could make *P. aeruginosa* more resistant to antibiotics.

Interestingly, the effects of tetracycline were unchanged in all three strains. The difference in sensitivity could be due to the difference in antibiotic resistance classifications (i.e., intrinsic, acquired, and adaptive). Resistance to tetracycline would fall under an intrinsic classification and would include outer membrane permeability, expression efflux pumps or production of antibiotic-inactivating enzyme, whereas the development of resistance to cefepime and ciprofloxacin would fall under adaptive resistance (Reygaert, 2018). One hypothesis is that this tolerance is linked to biofilm protection, which limits the access of the antibiotic. Since the Δ*pmtA* mutant has a weakened ability to form biofilms, this phenomenon could explain the increased sensitivity to these two therapeutics. Alternatively, pyocyanin levels have been linked to MDR *P. aeruginosa* strains, with higher levels being found in MDR vs. non-MDR strains (Gajdács et al., 2021). This suggests that the Δ*pmtA* mutant’s lower levels of pyocyanin could convey sensitivity, possibly due to a decrease in oxidant production.

The substantial decrease in pyocyanin levels and biofilm formation in the PmtA-deficient Δ*pmtA* strains suggests that this strain is less able to influence the host immune response and thus would be less able to establish a persistent infection. The results reported in the present study suggest that a deficiency in PmtA expression results in a significant change to *P. aeruginosa* virulence. We used *G. mellonella* larvae to assess the importance of PmtA in the innate immune response as a virulence factor. Our results showed the Δ*pmtA* mutant was 90% less virulent than wild-type PAO1. The virulence was restored in the complemented strain Δ*pmtA:pmtA*, indicating that PmtA is an important protein for establishing infection in a host. Taken together, these results indicate that PmtA can affect *P. aeruginosa* virulence by influencing pyocyanin expression.

It appears likely that therapeutic manipulation of PmtA during infection could decrease pyocyanin production and reduce biofilm formation. This disruption of PmtA may be an easier therapeutic target than interfering with the several genes involved in pyocyanin expression production. This work also demonstrates the complex regulation of pyocyanin, which is governed in part by PmtA expression. Since pyocyanin expression and biofilm formation could be rescued with an exogenous antioxidant addition, PmtA likely controls the transcription of the genes involved in pyocyanin expression by altering the redox environment. A change in the redox environment could also affect the expression of other proteins involved in *P. aeruginosa* virulence. The results of the present study show that PmtA affects the expression of virulence factors involved in establishing *P. aeruginosa* infection. Thus, targeting PmtA may represent a new, innovative option in treating the human pathogen *P. aeruginosa.*

## Data Availability Statement

The original contributions presented in the study are included in the article/Supplementary Material, further inquiries can be directed to the corresponding authors.

## Author Contributions

AT planned, performed the deletion and complementation of *pmtA*, bacterial growth curves, pyocyanin extraction, qPCR, biofilm assay, waxworm infection, and wrote the manuscript. KP planned, performed bacterial growth curves, pyocyanin extraction, biofilm assay, waxworm infections, and wrote the manuscript. ML planned, supervised the experiments, and helped wrote the manuscript. MM-M planned, performed acyl homoserine lactone-dependent QS assay, bacterial growth curves, MIC determination, and waxworm infections, and helped wrote the manuscript. CM performed bacterial growth curves, pyocyanin extractions, and GSH recovery experiments. JM planned deletion, complementation of mutants, and edit manuscript. JG planned and discussed the experiments. All authors contributed to the article and approved the submitted version.

## Conflict of Interest

The authors declare that the research was conducted in the absence of any commercial or financial relationships that could be construed as a potential conflict of interest.

## Publisher’s Note

All claims expressed in this article are solely those of the authors and do not necessarily represent those of their affiliated organizations, or those of the publisher, the editors and the reviewers. Any product that may be evaluated in this article, or claim that may be made by its manufacturer, is not guaranteed or endorsed by the publisher.

## Supplementary Material

Supplementary Figure S1 Schematic representation of *P. aeruginosa pmtA* knock-out strain construction. Schematic indicates the generation of the Δ*pmtA* mutant.

Supplementary Figure S2 Confirmation of Tn7 insertion into clean deletion mutant by colony PCR. Lanes 2, 3, and 4 used the pTn7GM_F and pTnGM_R primers, while lanes 5 and 6 used the PPA2140_Tn7_F and PA2140_Tn7_R primers with GoTaq Green Master Mix as previously described. Lane 1, 100 bp ladder; lane 2, pUC18R6K-mini-Tn7T-Gm-promoterPA2140 transformant (979 bp); lane 3, complemented Δ*pmtA* mutant (Δ*pmtA:pmtA*); lane 4, pUC18R6K-mini-Tn7T-Gm empty vector (261 bp); lane 5, wild-type PAO1 (781 bp); and lane 6, complemented Δ*pmtA* mutant Δ*pmtA:pmtA* (781 bp).

Supplementary Figure S3 *P. aeruginosa* strains PAO1, Δ*pmtA*, and Δ*pmtA:pmtA* grow at similar rates. Single colonies of the *P. aeruginosa* strains were each grown overnight in 5 mL of LB media at 37°C with vigorous shaking. These cultures were transferred to **(A)**. 50 mL LB media at a dilution of 1:100 and grown at 37°C with vigorous shaking. Growth was measured at 2, 4, 6, 24, and 30 h by removing 250 μL of culture in triplicate and transferring to a NUNC 96 well plate. The plate was measured using a Spectramax microplate reader. **(B)** 1 mL of 1:100 diluted cultures were transferred to a NUNC 96 well plate in triplicate. Growth was monitored over 15 h at 37°C with 5 s shaking every 5 min in a Spectramax microplate reader at OD_600_. **(C)** Cultures were grown in M9 salts media in a 24 well plate and monitored over 15 h at 37°C with 5 s shaking every 1 h in a Spectramax microplate reader at OD_600_. The data are presented as the average of three biological replicates (± standard error of the mean) and are representative of three separate experiments.

Supplementary Figure S4 PmtA is not required for survival in oxidative stress conditions. Overnight cultures were diluted at 1:100 and grown for 2 h. Cultures were adjusted to 0.08 OD at OD_600_. 600 μL of culture in triplicate were put in a 24-well plate containing either LB **(A)** or M9 salt media **(B)**. Hydrogen peroxide to a final concentration of 10 mM or water was added to the wells. **(A)** Growth was measured at OD_600_ every hour with shaking before and after measurement in a Spectramax microplate reader. The data are presented as the average of three biological replicates (± standard error of the mean) and are representative of three separate experiments.

Supplementary Figure S5 PmtA is not required for survival in heavy metal stress conditions. Overnight cultures were diluted at 1:100 and grown for 2 h in LB or M9 salt media. Cultures were adjusted to 0.08 OD at OD_600_. 600 μL of culture in triplicate were put in a 24-well plate. Zinc chloride **(A,B)** to a final concentration of 200 μM, or cadmium chloride **(C,D)** to a final concentration of 100 μM. Growth was measured at OD_600_ every hour with shaking before and after measurement in a Spectramax microplate reader. The data are presented as the average of three biological replicates (± standard error of the mean) and are representative of three separate experiments.

Supplementary Figure S6 PmtA does not play a role in iron uptake. Wild-type PAO1, Δ*pmtA*, and Δ*pmtA:pmtA* cultures were grown overnight in M9 salt media, diluted at 1:100 and grown in M9 salt media until they reached an OD_600_ of 0.08. 600 μL of culture in triplicate were put in a 24-well plate. M9 salt media (orange), EDDA to a final concentration of 150 μM (blue), EDDA + FeCl_2_ (black) to a final concentration of 150 μM and 50 μg, or EDDA + Transferrin (purple) to a final concentration of 150 μM and 200 μg. Growth was measured at OD_600_ every hour with shaking before and after measurement in a Spectramax microplate reader. The circles indicate PAO1, the squares indicate Δ*pmtA*, and the triangles indicate Δ*pmtA:pmtA.* The data are presented as the average of three biological replicates (± standard error of the mean) and are representative of three separate experiments.

Supplementary Table S1 Bacterial strains and plasmids used in the present study.

Supplementary Table S2 Primers used in the present study.
