# Supplementary figures and images for: PmtA Regulates Pyocyanin Expression and Biofilm Formation in Pseudomonas aeruginosa
Source: Front Microbiol. 2021 Nov 15;12:789765. doi: 10.3389/fmicb.2021.789765 (PMC8636135; doi:10.3389/fmicb.2021.789765)

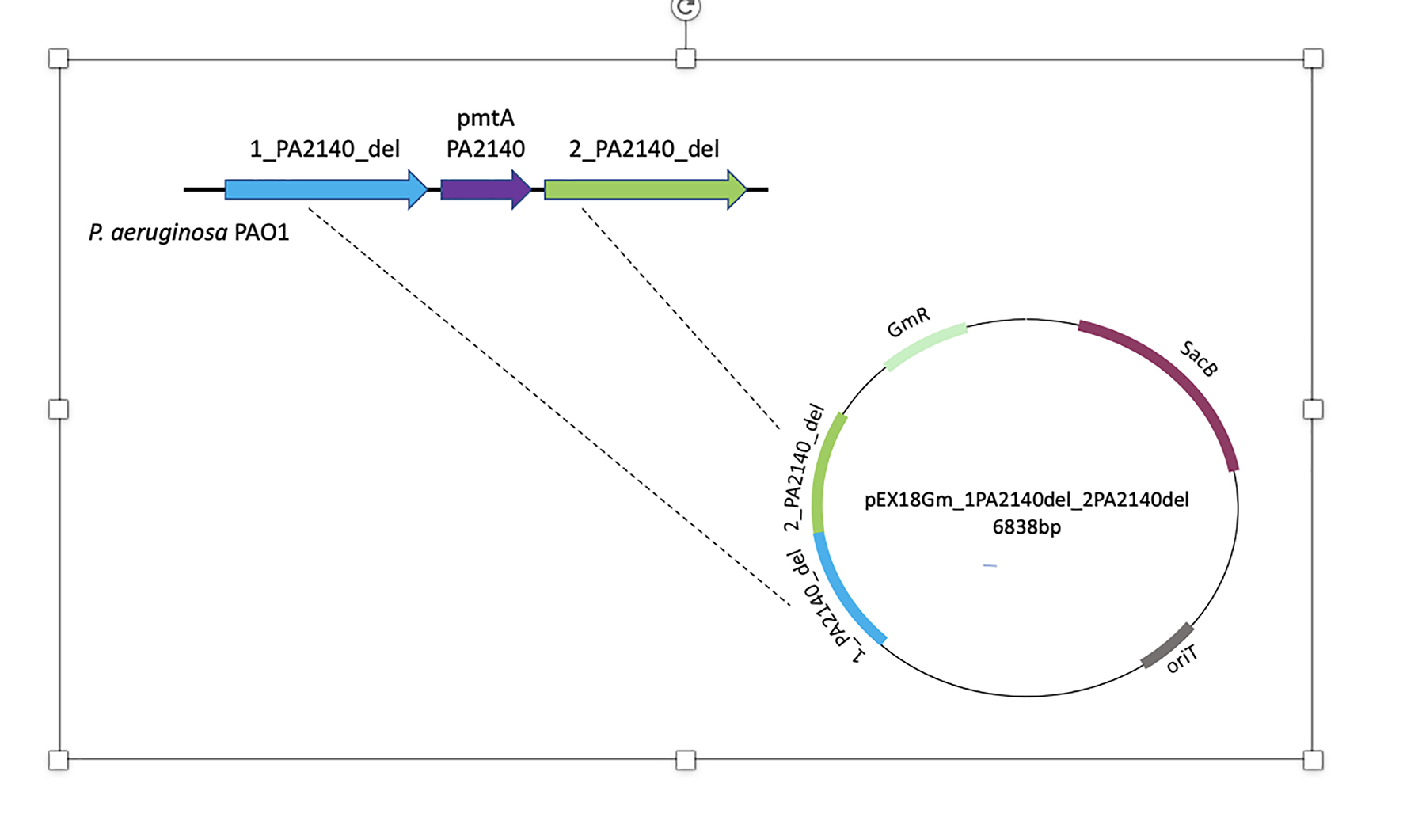

Supplement: Supplementary Figure S1 — Schematic representation of P. aeruginosa pmtA knock-out strain construction. Schematic indicates the generation of the ΔpmtA mutant. [file Image_1.TIF]

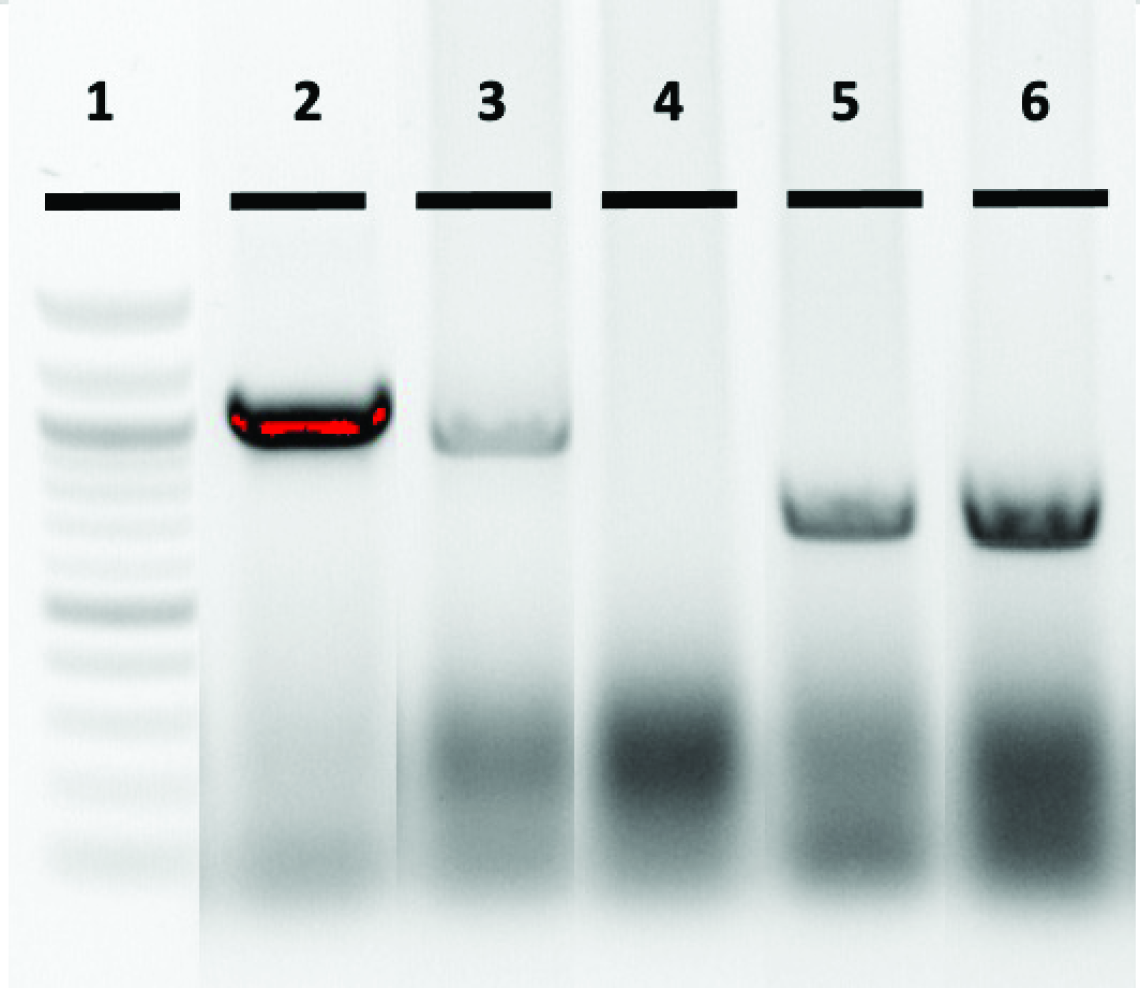

Supplement: Supplementary Figure S2 — Confirmation of Tn7 insertion into clean deletion mutant by colony PCR. Lanes 2, 3, and 4 used the pTn7GM_F and pTnGM_R primers, while lanes 5 and 6 used the PPA2140_Tn7_F and PA2140_Tn7_R primers with GoTaq Green Master Mix as previously described. Lane 1, 100 bp ladder; lane 2, pUC18R6K-mini-Tn7T-Gm-promoterPA2140 transformant (979 bp); lane 3, complemented ΔpmtA mutant (ΔpmtA:pmtA); lane 4, pUC18R6K-mini-Tn7T-Gm empty vector (261 bp); lane 5, wild-type PAO1 (781 bp); and lane 6, complemented ΔpmtA mutant ΔpmtA:pmtA (781 bp). [file Image_2.TIF]

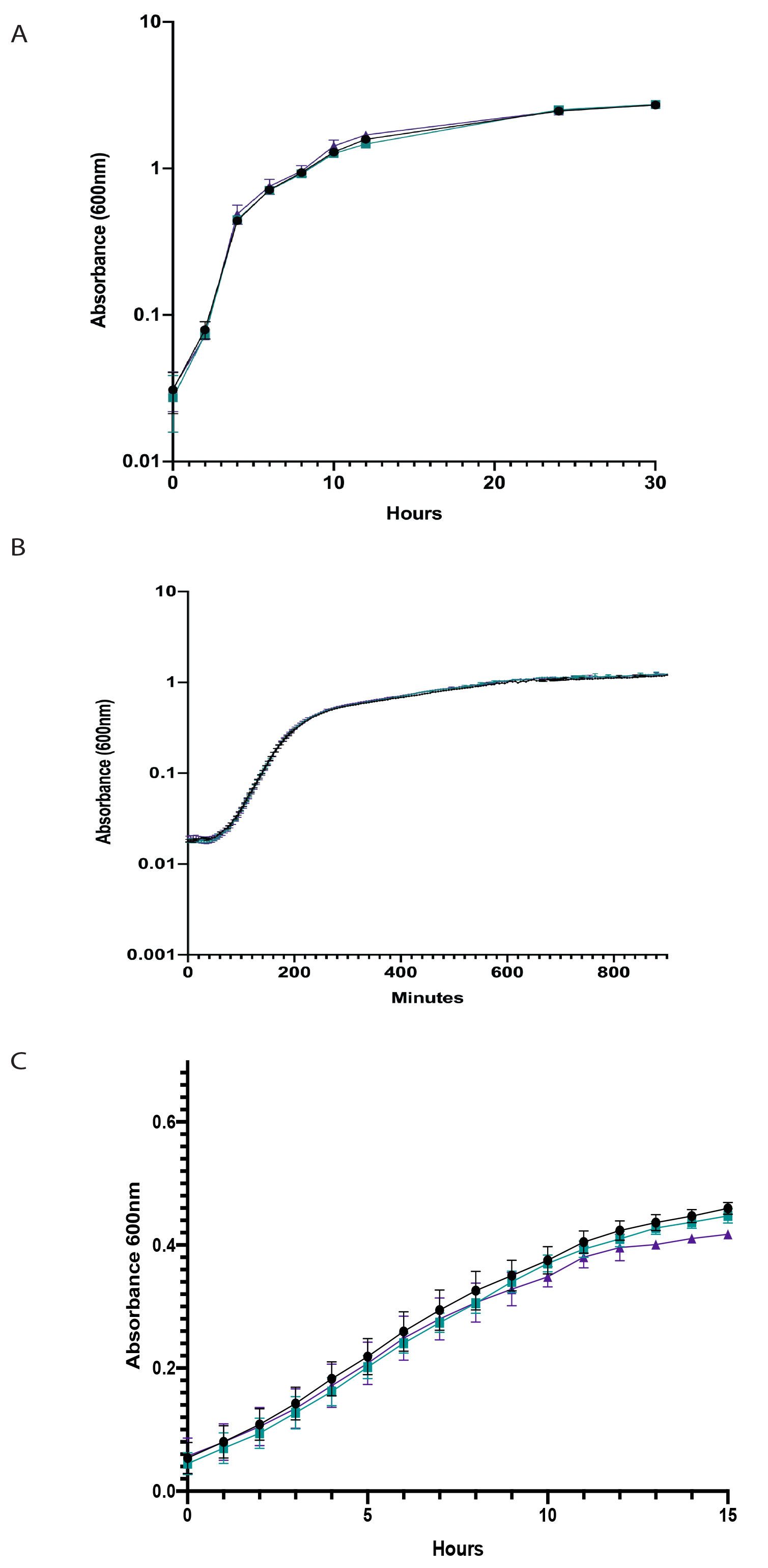

Supplement: Supplementary Figure S3 — P. aeruginosa strains PAO1, ΔpmtA, and ΔpmtA:pmtA grow at similar rates. Single colonies of the P. aeruginosa strains were each grown overnight in 5 mL of LB media at 37°C with vigorous shaking. These cultures were transferred to (A). 50 mL LB media at a dilution of 1:100 and grown at 37°C with vigorous shaking. Growth was measured at 2, 4, 6, 24, and 30 h by removing 250 μL of culture in triplicate and transferring to a NUNC 96 well plate. The plate was measured using a Spectramax microplate reader. (B) 1 mL of 1:100 diluted cultures were transferred to a NUNC 96 well plate in triplicate. Growth was monitored over 15 h at 37°C with 5 s shaking every 5 min in a Spectramax microplate reader at OD600. (C) Cultures were grown in M9 salts media in a 24 well plate and monitored over 15 h at 37°C with 5 s shaking every 1 h in a Spectramax microplate reader at OD600. The data are presented as the average of three biological replicates (± standard error of the mean) and are representative of three separate experiments. [file Image_3.TIF]

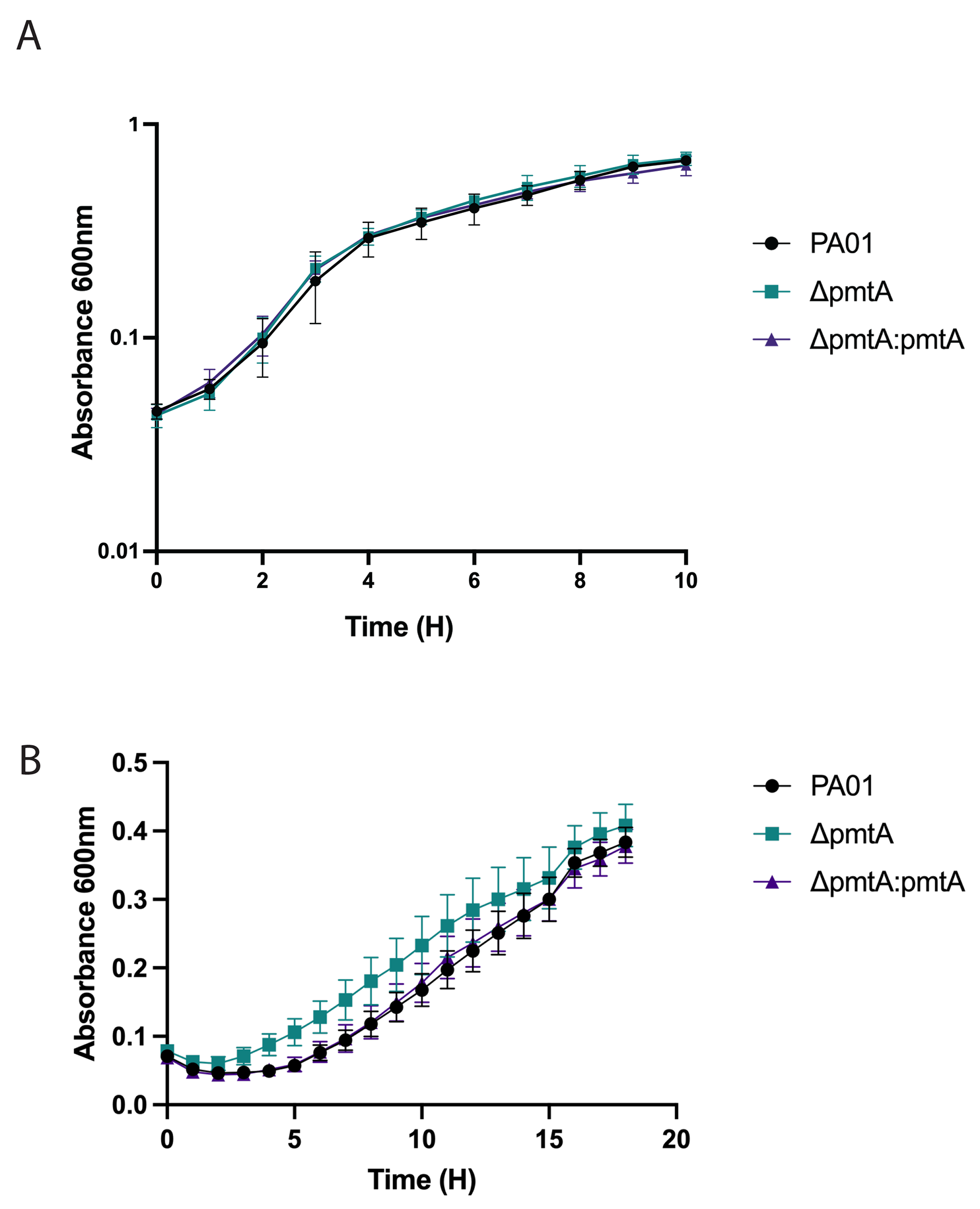

Supplement: Supplementary Figure S4 — PmtA is not required for survival in oxidative stress conditions. Overnight cultures were diluted at 1:100 and grown for 2 h. Cultures were adjusted to 0.08 OD at OD600. 600 μL of culture in triplicate were put in a 24-well plate containing either LB (A) or M9 salt media (B). Hydrogen peroxide to a final concentration of 10 mM or water was added to the wells. (A) Growth was measured at OD600 every hour with shaking before and after measurement in a Spectramax microplate reader. The data are presented as the average of three biological replicates (± standard error of the mean) and are representative of three separate experiments. [file Image_4.TIF]

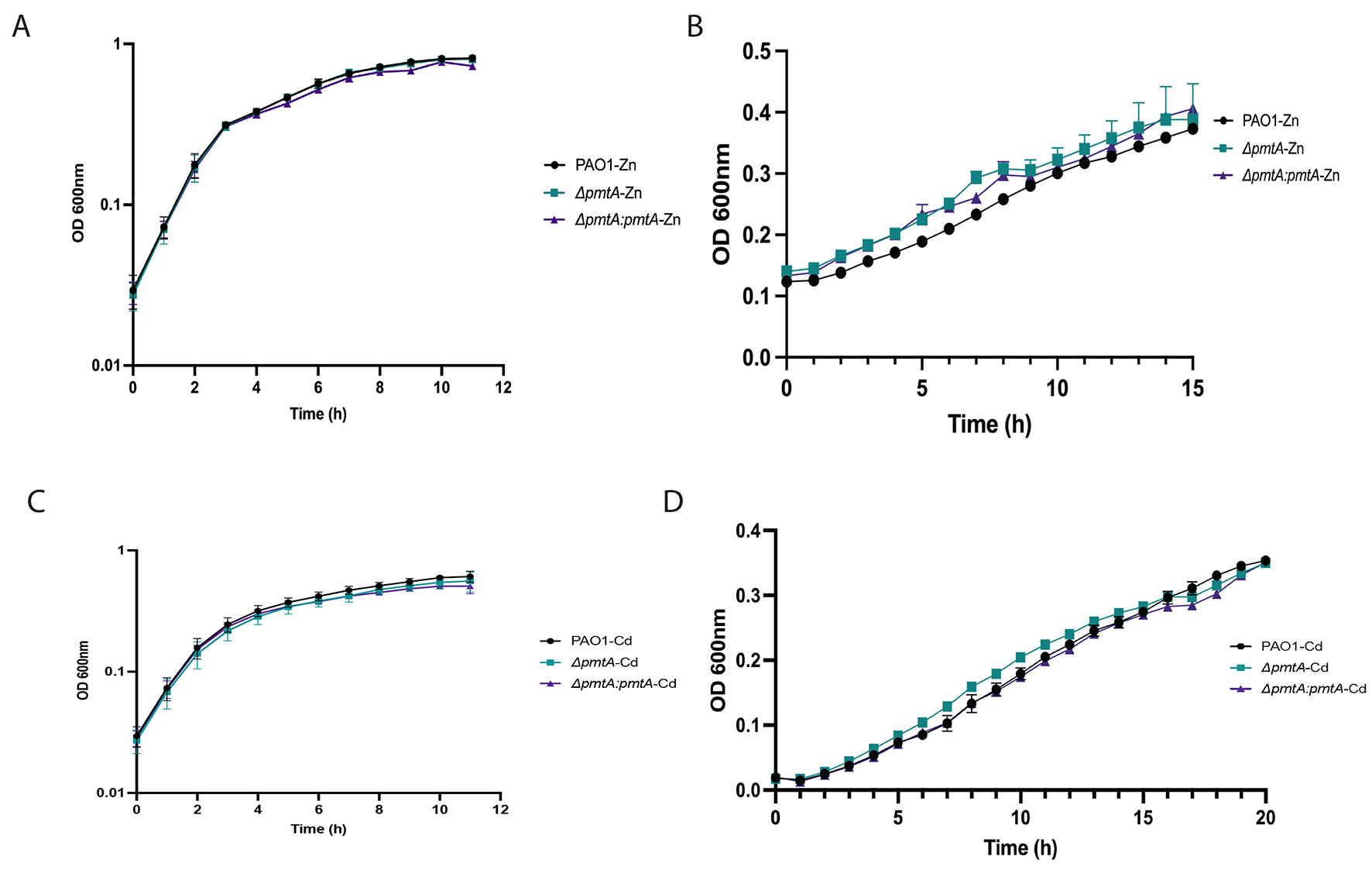

Supplement: Supplementary Figure S5 — PmtA is not required for survival in heavy metal stress conditions. Overnight cultures were diluted at 1:100 and grown for 2 h in LB or M9 salt media. Cultures were adjusted to 0.08 OD at OD600. 600 μL of culture in triplicate were put in a 24-well plate. Zinc chloride (A,B) to a final concentration of 200 μM, or cadmium chloride (C,D) to a final concentration of 100 μM. Growth was measured at OD600 every hour with shaking before and after measurement in a Spectramax microplate reader. The data are presented as the average of three biological replicates (± standard error of the mean) and are representative of three separate experiments. [file Image_5.TIF]

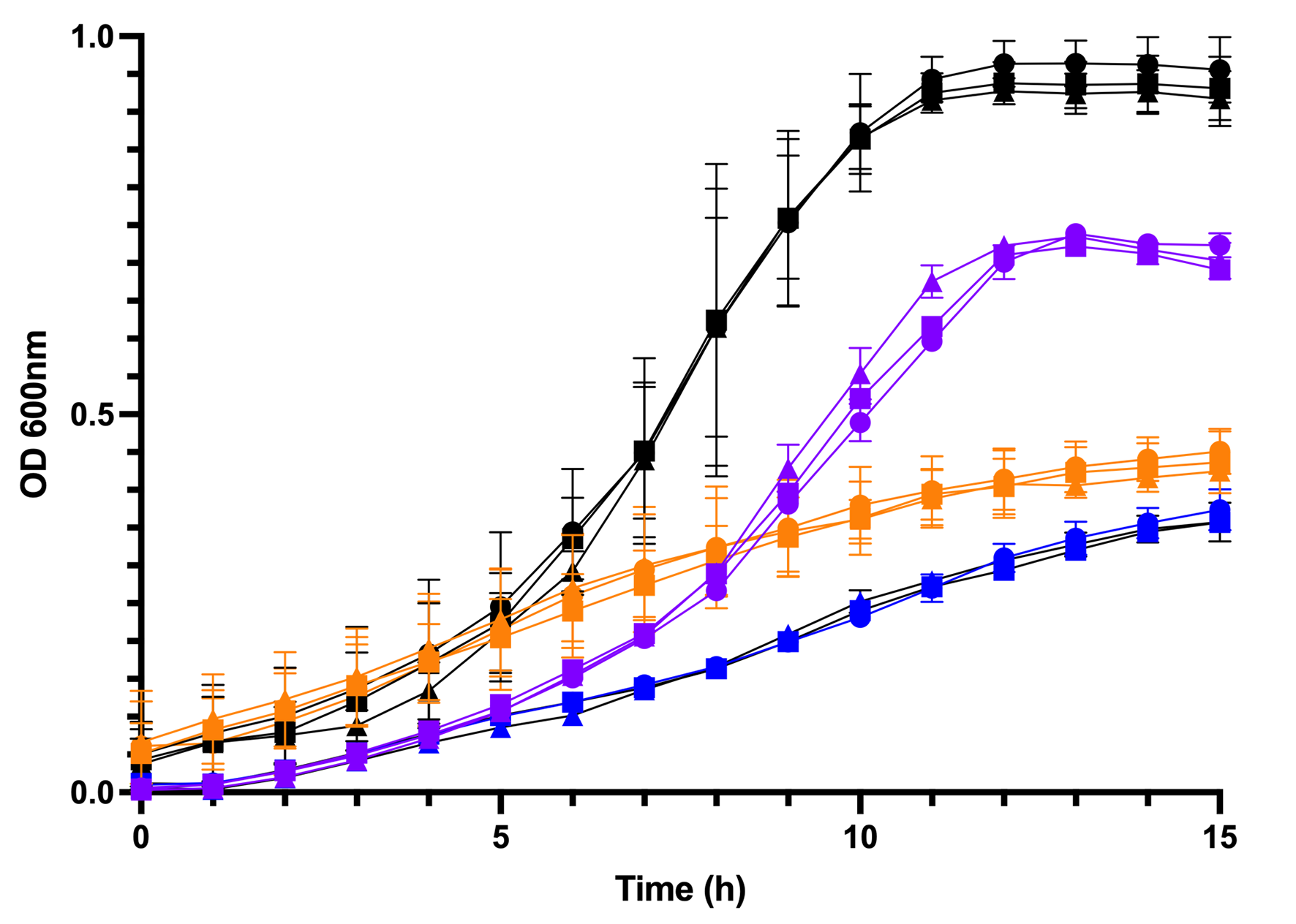

Supplement: Supplementary Figure S6 — PmtA does not play a role in iron uptake. Wild-type PAO1, ΔpmtA, and ΔpmtA:pmtA cultures were grown overnight in M9 salt media, diluted at 1:100 and grown in M9 salt media until they reached an OD600 of 0.08. 600 μL of culture in triplicate were put in a 24-well plate. M9 salt media (orange), EDDA to a final concentration of 150 μM (blue), EDDA + FeCl2 (black) to a final concentration of 150 μM and 50 μg, or EDDA + Transferrin (purple) to a final concentration of 150 μM and 200 μg. Growth was measured at OD600 every hour with shaking before and after measurement in a Spectramax microplate reader. The circles indicate PAO1, the squares indicate ΔpmtA, and the triangles indicate ΔpmtA:pmtA. The data are presented as the average of three biological replicates (± standard error of the mean) and are representative of three separate experiments. [file Image_6.TIF]
