# Supplementary material for: PmtA Regulates Pyocyanin Expression and Biofilm Formation in Pseudomonas aeruginosa
Source: Front Microbiol. 2021 Nov 15;12:789765. doi: 10.3389/fmicb.2021.789765 (PMC8636135; doi:10.3389/fmicb.2021.789765)
Supplement: Supplementary Table S1 — Bacterial strains and plasmids used in the present study. [file Table_1.docx]

**Supplemental Table S1 Bacterial strains and plasmids used in the present study**

| **Bacterial strains or plasmids** | **Characteristics** | **Source or reference** |
| --- | --- | --- |
| ***Escherichia coli* strains** |  |  |
| Pir1 | F*^-^ ∆lac169 rpoS(Am) robA1 creC510 hsdR514* endA recA1 uidA(∆MluI)::pir-116 | Invitrogen |
| MG4 | pKDT17 | Addgene |
| ***Pseudomonas aeruginosa s*trains** |  |  |
| PAO1 | Wild-type | Manoil Lab UW |
| ∆pmtA | PAO1*∆*PA2140 | This study |
| pmtA:pmtA | PAO1, *∆*PA2140, Gm^r^ | This study |
| **Plasmids** |  |  |
| pEX18Gm  AF047518.1 | Gene replacement vector; *sacB*; Gm^r^ | Schweizer lab |
| pUC18R6K-mini-Tn7T-Gm  DQ153108.1 | Gene replacement vector; Gm^r^ | Addgene |
| pGEX-6p-*pmtA* | pGEX-6p-1, PA2140 | This study |

**Supplemental Table S2 Primers used in the present study**

| Primer | Sequence |
| --- | --- |
| Deletion of PA2140 |  |
| 1_PA2140_del_F | TGCAGGTCGACTCTAGAGGTGTCGGTACCGGTGTTC |
| 1_PA2140_del_R | GTTCATGGGCAGTCTCCAG |
| 2_PA2140_del_F | TGGAGACTGCCCATGAACATCTCGCCCTGAGGAGCG |
| 2_PA2140_del_R | CAGCTATGACCATGATTACGTTGATGGAATTCGGGCAG |
| Screen for inserts |  |
| 2_PA2140_test_F | CTGCTGATCCTGCTCCTG |
| 2_PA2140_test_R | CAGTTCGGCGCTGATCTG |
| Sequencing |  |
| PA2140_Tn7_F | ATGAACAGCGAAACCTGTGC |
| PA2140_Tn7_R | GGCCTTCGCGAGGTACGCTCCTCAGGGCGAGATC |
| Cloning of PA2140 |  |
| PPA2140_Tn7_F | TGGATCCCCCGGGCTGCAAACCAATGGAACCTGCGC |
| PA2140_Tn7_R | GGCCTTCGCGAGGTACGCTCCTCAGGGCGAGATC |
| Sequencing |  |
| pTn7GM_F | TGCTGTTGACAAAGGGAATC |
| pTn7GM_R | GTAGCGTCGTAAGCTAATACG |
| RT-PCR |  |
| qPCR_PmtA_F | ACCTGTGCCTGTCCCAAAT |
| qPCR_PmtA_R | CGGGAAAGGTCTCCTTCAAC |
| qPCR_RNApol_F | TGATTTCGGTCAGGGACTTC |
| qPCR_RNApol_R | GATGACCTGGAACTGACCGT |
| qPCR_phzH_F | TTATCCGACTTCTGCCAACC |
| qPCR_phzH_R | GAGAGCCCGTACAACCTGAG |
| qPCR_phzM_F | CAAGTTGTTACCGGGGAATG |
| qPCR_phzM_R | AGATCTCGAAGGCCACCAG |

**Figure S1 Schematic representation of *P. aeruginosa pmtA* knock-out strain construction.** Schematic indicates the generation of the *ΔpmtA* mutant.

Figure S2. Confirmation of Tn7 insertion into clean deletion mutant by colony PCR Lanes 2, 3, and 4 used the pTn7GM_F and pTnGM_R primers, while lanes 5 and 6 used the PPA2140_Tn7_F and PA2140_Tn7_R primers with GoTaq Green Master Mix as previously described. Lane 1, 100 bp ladder; lane 2, pUC18R6K-mini-Tn7T-Gm-promoterPA2140 transformant (979 bp); lane 3, complemented *ΔpmtA* mutant (*ΔpmtA:pmtA*); lane 4, pUC18R6K-mini-Tn7T-Gm empty vector (261 bp); lane 5, wild-type PAO1 (781 bp); and lane 6, complemented *ΔpmtA* mutant *ΔpmtA:pmtA*, (781 bp)

**Figure S3.** *P. aeruginosa* strains PAO1, *ΔpmtA,* and *ΔpmtA:pmtA* grow at similar rates**.** Single colonies of the *P. aeruginosa* strains were each grown overnight in 5 mL of LB media at 37°C with vigorous shaking. These cultures were transferred to **A.** 50 mL LB media at a dilution of 1:100 and grown at 37°C with vigorous shaking. Growth was measured at 2, 4, 6, 24, and 30 hours by removing 250 μL of culture in triplicate and transferring to a NUNC 96 well plate. The plate was measured using a Spectramax microplate reader. **B.** 1 mL of 1:100 diluted cultures were transferred to a NUNC 96 well plate in triplicate. Growth was monitored over 15 hours at 37°C with 5s shaking every 5 min in a Spectramax microplate reader at OD_600_. C. Cultures were grown in M9 salts media in a 24 well plate and monitored over 15 hours at 37°C with 5s shaking every 1 hour in a Spectramax microplate reader at OD_600_. The data are presented as the average of three biological replicates (+/- standard error of the mean) and are representative of three separate experiments.

### Figure S4. PmtA is not required for survival in oxidative stress conditions.

Overnight cultures were diluted at 1:100 and grown for 2 hours. Cultures were adjusted to 0.08 OD at OD_600_. 600 μL of culture in triplicate were put in a 24-well plate containing either LB (A) or M9 salt media (B). Hydrogen peroxide to a final concentration of 10 mM or water was added to the wells. **A.** Growth was measured at OD_600_ every hour with shaking before and after measurement in a Spectramax microplate reader. The data are presented as the average of three biological replicates (+/- standard error of the mean) and are representative of three separate experiments.

Figure S5. PmtA is not required for survival in heavy metal stress conditions. Overnight cultures were diluted at 1:100 and grown for 2 hours in LB or M9 salt media. Cultures were adjusted to 0.08 OD at OD_600_. 600 μL of culture in triplicate were put in a 24-well plate. Zinc chloride (A&B) to a final concentration of 200 μM, or cadmium chloride (C&D) to a final concentration of 100 μM. Growth was measured at OD_600_ every hour with shaking before and after measurement in a Spectramax microplate reader. The data are presented as the average of three biological replicates (+/- standard error of the mean) and are representative of three separate experiments.

**Figure S6. PmtA does not play a role in iron uptake.** Wild-type PAO1, *ΔpmtA,* and *ΔpmtA:pmtA* cultures were grown overnight in M9 salt media, diluted at 1:100 and grown in M9 salt media until they reached an OD_600_ of 0.08. 600μL of culture in triplicate were put in a 24-well plate. M9 salt media (orange), EDDA to a final concentration of 150μM (Blue), EDDA + FeCl_2_ (Black) to a final concentration of 150μM and 50μg, or EDDA+ Transferrin (purple) to a final concentration of 150μM and 200μg. Growth was measured at OD_600_ every hour with shaking before and after measurement in a Spectramax microplate reader. The circles indicate PAO1, the squares indicate *ΔpmtA,* and the triangles indicate *ΔpmtA:pmtA.* The data are presented as the average of three biological replicates (+/- standard error of the mean) and are representative of three separate experiments
